# Effect Modifying Role of Serum Calcium on Mortality-Predictability of PTH and Alkaline Phosphatase in Hemodialysis Patients: An Investigation Using Data from the Taiwan Renal Registry Data System from 2005 to 2012

**DOI:** 10.1371/journal.pone.0129737

**Published:** 2015-06-24

**Authors:** Yen-Chung Lin, Yi-Chun Lin, Chiao-Ying Hsu, Chih-Chin Kao, Fan-Chi Chang, Tzen-Wen Chen, Hsi-Hsien Chen, Chi-Cheng Hsu, Mai-Szu Wu, Taiwan Society of Nephrology

**Affiliations:** 1 Division of Nephrology, Department of Internal Medicine, Taipei Medical University Hospital, Taipei, Taiwan; 2 Department of Internal Medicine, School of Medicine, College of Medicine, Taipei Medical University, Taipei, Taiwan; 3 Graduate Institute of Clinical Medicine, College of Medicine, Taipei Medical University, Taipei, Taiwan; 4 Division of Endocrinology & Metabolism, Department of Medicine, Taipei Veterans General Hospital, Taipei, Taiwan; 5 Faculty of Medicine, National Yang-Ming University, Taipei, Taiwan; 6 Center for Health Policy Research and Development, National Health Research Institutes, Miaoli, County, Taiwan; Mario Negri Institute for Pharmacological Research and Azienda Ospedaliera Ospedali Riuniti di Bergamo, ITALY

## Abstract

Predicting mortality in dialysis patients based on low intact parathyroid hormone levels is difficult, because aluminum intoxication, malnutrition, older age, race, diabetes, or peritoneal dialysis may influence these levels. We investigated the clinical implications of low parathyroid hormone levels in relation to the mortality of dialysis patients using sensitive, stratified, and adjusted models and a nationwide dialysis database. We analyzed data from 2005 to 2012 that were held on the Taiwan Renal Registry Data System, and 94,983 hemodialysis patients with valid data regarding their intact parathyroid levels were included in this study. The patient cohort was subdivided based on the intact parathyroid hormone and alkaline phosphatase levels. The mean hemodialysis duration within this cohort was 3.5 years. The mean (standard deviation) age was 62 (14) years. After adjusting for age, sex, diabetes, the hemodialysis duration, serum albumin levels, hematocrit levels, calcium levels, phosphate levels, and the hemodialysis treatment adequacy score, the single-pool Kt/V, the crude and adjusted all-cause mortality rates increased when alkaline phosphatase levels were higher or intact parathyroid hormone levels were lower. In general, at any given level of serum calcium or phosphate, patients with low intact parathyroid hormone levels had higher mortality rates than those with normal or high iPTH levels. At a given alkaline phosphatase level, the hazard ratio for all-cause mortality was 1.33 (p < 0.01, 95% confidence interval 1.27–1.39) in the group with intact parathyroid hormone levels < 150 pg/mL and serum calcium levels > 9.5 mg/dL, but in the group with intact parathyroid hormone levels > 300 pg/mL and serum calcium levels > 9.5 mg/dL, the hazard ratio was 0.92 (95% confidence interval 0.85–1.01). Hence, maintaining albumin-corrected high serum calcium levels at > 9.5 mg/dL may correlate with poor prognoses for patients with low intact parathyroid hormone levels.

## Introduction

Chronic kidney disease (CKD)-mineral and bone disorder (MBD) increases morbidity and mortality in end-stage renal disease (ESRD) patients.[1] Renal osteodystrophy may be initiated at the onset of CKD because of an increase in urinary phosphate (P) excretion. However, low parathyroid hormone (PTH) levels have long been associated with aluminum intoxication, malnutrition, older age, race, diabetes, peritoneal dialysis (PD), calcium (Ca)-containing P binders, and vitamin D overuse.[2,3] Therefore, it is difficult to determine the true association between low PTH levels and mortality.

Vascular calcification may be associated with low PTH levels in ESRD patients,[4,5] and coronary artery disease is the leading cause of death in these patients. However, normal PTH levels do not prevent low turnover disease.[6] In addition, low PTH levels may be linked to hypercalcemia.[7] Hypercalcemia may increase the mortality of ESRD patients with abnormal PTH levels at any given serum P level.[8] Therefore, risk stratification of patients based on the Ca or P levels as well as the PTH levels is crucial.[9]

In this study, we analyzed data from a large, nationwide, and established ESRD registration database, the Taiwan Renal Registry Data System (TWRDS),[10] to determine predictors of mortality that included PTH, serum Ca, and P levels.

## Materials and Methods

This study was approved by the ethics committee of Taipei Medical University’s institutional review board (Number: 201411017), and was conducted in accordance with the principles of the Declaration of Helsinki. The requirement for written informed consent was waived.

### The Taiwan Renal Registry Data System

The TWRDS was initially established in 1987 for the accreditation of dialysis therapy. All dialysis units in Taiwan must provide the appropriate information for inclusion in the TWRDS to obtain national health insurance (NHI)-associated reimbursements. Every dialysis unit submits a quarterly report. In 1996, a self-developed software program, HOPE, was used for computerized data collection. In 1997, additional data were gathered, including those relating to co-morbidities, the patients’ rehabilitation statuses, and the dialysis adequacy indices, which included the biochemical and hematological parameters, the hepatitis serological results, hypertension control, anemia management, and mineral bone indices.[11] The data within the TWRDS provide a robust foundation for the continual quality control of dialysis at the national level.[12–15] We analyzed the data in the TWRDS database that were generated between 2005 and 2012. During this period, 119,115 dialysis patients were registered within the database.

### Patient enrollment

By December 31, 2011, 569 hemodialysis (HD) units and 117 PD units had been established in Taiwan, an increase of 15 and 17 units, respectively, compared with the numbers of these units in December 2008. The dialysis units submit quarterly and annual reports to the TWRDS, and the data we obtained from these reports are described next.

**Fig 1:**
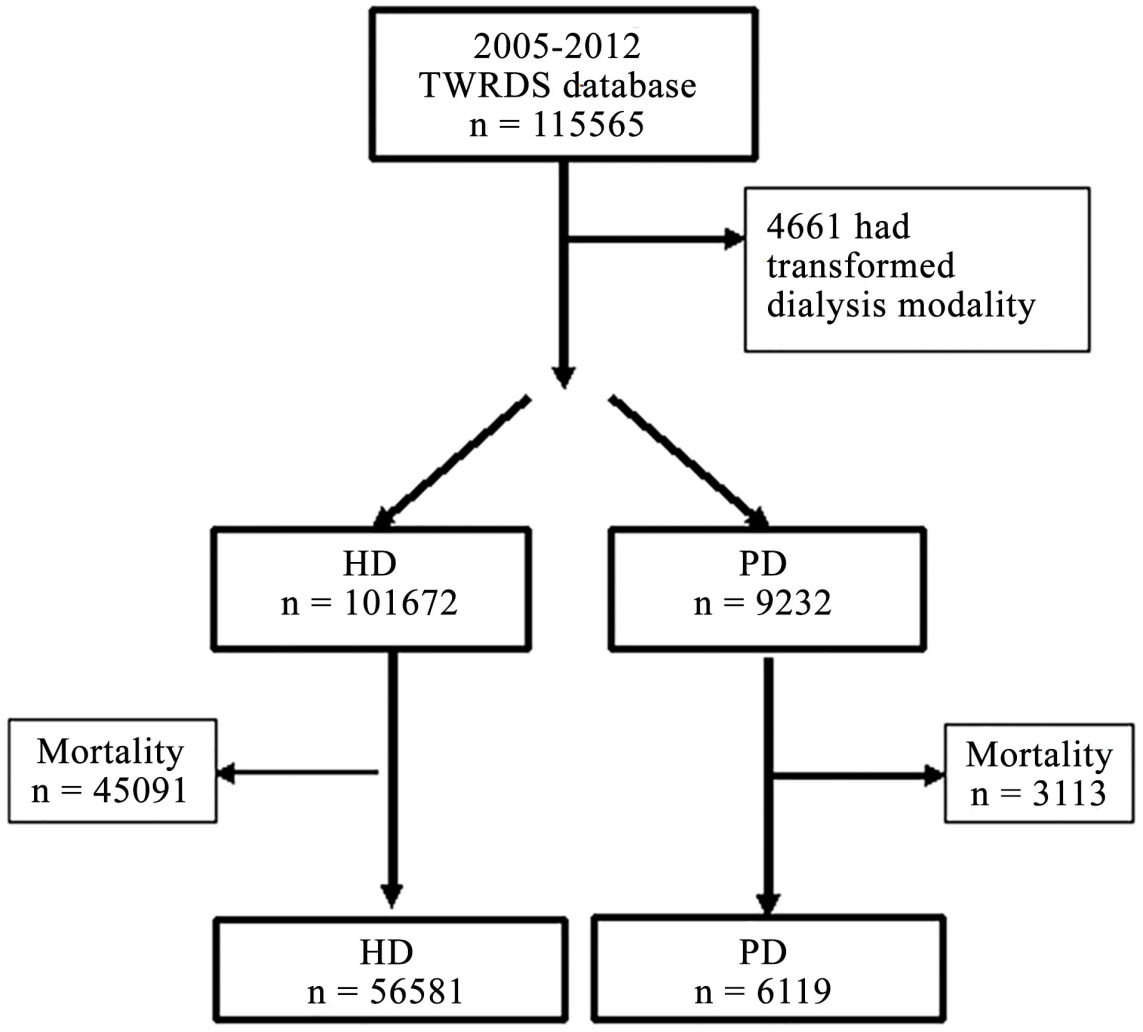
Flow diagram of the study cohort. The study’s data were derived from patients who were registered on the Taiwan Renal Registry Data System between 2005 and 2012.

Patients registered with the TWRDS from 2005 to 2012 were included in the analysis (n = 115,565). Patients who had received HD or PD for > 1 month were assigned to either the HD group or the PD group, respectively. After excluding 4,661 patients who had changed their dialysis modality, the final sample that went forward for analysis comprised 110,994 patients (Fig 1). Of these patients, 101,672 patients (91.6%) opted for HD and 9,232 patients (8.3%) opted for PD as their initial renal replacement therapy (RRT) modality between 2005 and 2012 (Fig 1). Among the HD patients, 6,113 (6%) had data missing in relation to their intact PTH (iPTH) levels, and 586 (0.6%) had data missing in relation to their alkaline phosphatase (ALP) levels; therefore, 94,983 patients were included in this analysis. Most patients were lost to follow up because of death, which was determined based on the complete national coverage provided by the NHI policy for all RRT expenditure. Most of the hospitals in Taiwan (97.9%) for intact PTH analysis was sent to the Union Clinical Laboratory, where second generation PTH Chemiluminescence assay ADVIA Centaur (Siemens Healthcare Diagnostics, Tarrytown, NY) was employed since 2007 (normal range 14–72 pg/mL). KDIGO guidelines on CKD-MBD were published in 2009 with second-generation intact PTH Allegro Nichols IRMA, and the commercial assays on average showed 60%–152% variation compared with the standard Nichols Allegro IRMA,[16] Therefore, the Union Clinical Laboratory still reported original data to the “HOPE” database of TWRDS.

### Statistical analysis

The descriptive statistics were expressed as the means (standard deviations [SD]), medians (ranges), or frequencies (percentages). The average PTH, Ca, P, and ALP levels were used. Serum Ca levels were corrected using the following formula: corrected *Ca* = (0.8 × [normal *albumin* level − the patient's *albumin level*]) + serum Ca. We performed Cox regression analysis to estimate the hazard ratios (HR) for PTH or ALP. The case-mix adjusted model included the following confounding factors: age, sex, the presence of diabetes mellitus, and the laboratory data, which included hematocrit levels, albumin levels, Ca levels, P levels, and the HD treatment adequacy score, the single-pool Kt/V. Low PTH levels were defined as those that were lower than the KDIGO-recommended PTH level, which is between two- and nine-times the upper limit of the normal PTH level. Since the upper limit for PTH in our assay was 72 pg/mL, we chose 150 pg/mL as the cutoff value for a low PTH level. Although the KDIGO guidelines suggest regular ALP measurements, a target range has not been specified. Hence, we used an ALP cutoff value of 120 U/L, because a higher ALP level correlated with mortality in a study from the University of California, Los Angeles (UCLA) study group that comprised a large population of HD patients.[17] In addition, another study found that an ALP level of ≥ 120 U/L was a robust predictor of coronary artery calcification.[18] All of the descriptive and multivariate analyses were performed using the Statistical Package for the Social Sciences software version 17.0 for Windows XP (SPSS Inc., Chicago, IL, USA) and SAS version 9.1 (SAS Institute, Cary, NC).

## Results

### Population demographics

Table 1 presents the study population’s demographic data. A total of 94,983 HD patients with valid data relating to iPTH and ALP levels were included in this study. The mean duration of HD in this cohort was 3.5 years. The mean (SD) age of the patients was 62 (14) years. Type 2 diabetes was present in 51% of the patients. We divided each categories of iPTH by a cut-off value of ALP 120 U/L. There were no obvious difference in characters between different ALP levels in the same iPTH group except the percentage ALP ≥ 120 U/L group was higher in the iPTH > 300 pg/mL group. Patients with high iPTH levels (≥ 300 pg/mL) tended to have longer dialysis durations, and higher serum P and ALP levels (Table 1). Patients with low iPTH levels (< 150 pg/mL) had low albumin and hematocrit levels, were older, and tended to have diabetes.

**Table 1:** Baseline characteristics of the patients according to the intact parathyroid hormone and Alkaline Phosphatase categories.

Variable	WholeGroup	i-PTH						P value
		<150 pg/mL		150–300 pg/mL		>300 pg/mL		
		ALP <120 U/L	ALP ≧120 U/L	ALP <120 U/L	ALP ≧120 U/L	ALP <120 U/L	ALP ≧120 U/L	
Number (%)	94983	30610(32.2%)	12137(12.8%)	18814(19.8%)	8397(8.8%)	13865(14.6%)	11160(11.7%)	
Age (years)	62 ± 14	65 ± 13	66 ± 12	61 ± 14	62 ± 13	56 ± 14	58 ± 14	<0.001
Male (%)	47499(50%)	15875(52%)	6108(50%)	9927(53%)	3753(45%)	7187(52%)	4649(42%)	<0.001
Type 2diabetes (%)	48228(51%)	18124(59%)	7202(59%)	9386(50%)	4287(51%)	5016(36%)	4213(38%)	<0.001
HD duration(years)	3.5	2.7	2.3	3.7	3.5	4.7	5.0	<0.001
**Laboratorydata**								
Albumin(g/dl)	3.8 ± 0.4	3.7 ± 0.4	3.6 ± 0.4	3.9 ± 0.3	3.8 ± 0.4	3.9 ± 0.3	3.9 ±0.3	<0.001
Hemotocrit(%)	31 ±3.1	30.9 ±2.9	30.3 ±3.2	31.3 ±3.0	30.9 ±3.3	31.6 ±3.3	31.3 ±3.4	<0.001
Calcium(mg/dL)	9.2 ±0.7	9.2 ±0.6	9.2 ±0.7	9.1 ±0.6	9.1 ±0.6	9.4 ±0.8	9.5 ±0.8	<0.001
Phosphate(mg/dL)	4.8 ±1.1	4.5 ±1.0	4.3 ±1.0	4.9 ±1.0	4.7 ±1.0	5.5 ±1.0	5.3 ±1.0	<0.001
CaP product	44.4 ±10.9	41.5 ±9.9	39.3 ±10.3	44.7 ±9.7	42.4 ±10.1	51.3 ±10.1	50.2 ±10.8	<0.001

iPTH: intact parathyroid hormone; ALP: alkaline phosphatase.

### Serum calcium and phosphate levels and their correlations with death

The risk of death increased when the serum Ca level reached 9.5 mg/dL (HR 1.05, 95% confidence interval [CI] 1.02–1.08) or the serum P level reached 5.5 mg/dL (HR 1.15, 95% CI 1.12–1.19). The risk of death also increased when the serum Ca levels were < 8.5 mg/dL or the serum P levels were < 3.5 mg/dL (Table 2).

**Table 2:** Risk of death within the different categories of serum calcium and phosphate levels.

	Number ofpatients withdata	%	Crude HR(95% confidenceinterval)	Adjusted HR(95% confidenceinterval)
**Calcium (mg/dL)**				
**< 8.5**	13,432	14	2.11 (2.05–2.17)**	1.41 (1.36–1.45)**
**8.5–9.5**	53,942	55	Reference	Reference
**9.5–10.5**	27,975	28	0.82 (0.80–0.84)**	1.05 (1.02–1.08)**
**> 10.5**	3,459	4	1.38 (1.32–1.44)**	1.77 (1.68–1.86)**
**Phosphate (mg/dL)**				
**< 3.5**	11,462	11	2.33 (2.27–2.39)**	1.19 (1.15–1.22)**
**3.5–5.5**	64,475	65	Reference	Reference
**5.5–6.5**	17,754	18	0.82 (0.79–0.84)**	1.15 (1.12–1.19)**
**6.5–7.5**	4713	5	1.04(0.99–1.09)	1.53(1.45–1.62)**
**7.5–8.5**	1,098	1	1.57 (1.44–1.72)**	(1.80–2.20)**
**> 8.5**	311	0	2.36 (2.02–2.77)**	2.46 (2.03–2.98)**

*p < 0.05

**p < 0.01 adjusted for age, sex, diabetes, hematocrit, albumin, kt/V.

HR: hazard ratio.

### Hazard ratio of all-cause mortality between different groups of calcium and phosphate and intact parathyroid hormone

Table 3 shows that the risk of all-cause mortality was highest in the group with low iPTH, high Ca, and low P levels (HR 2.62, 95% CI 2.43–2.81), while the risk of all-cause mortality was lowest in the group with high iPTH, high Ca, and normal P levels (HR 0.86, 95% CI 0.81–0.91). In general, those patients with low iPTH levels had higher mortality rates than those with normal or high iPTH levels at any given serum level of Ca or P.

**Table 3:** Different combination of categories of Ca/P/iPTH and the all-cause mortality in TWRDS.

Group	Number	%	Crude HR	Adjust HR
Total	94546	100%		
Normal i-PTH and Normal Ca and Normal P	11875	13%	Reference	Reference
High i-PTH and Normal Ca and Normal P	5422	6%	0.89(0.82–0.95)**	0.93(0.87–0.98)*
Low i-PTH and Normal Ca and Normal P	18708	20%	1.59(1.54–1.66)**	1.35(1.30–1.40)**
Normal i-PTH and Normal Ca and High P	3382	4%	1.11(1.03–1.82)**	1.31(1.23–1.41)**
Normal i-PTH and High Ca and High P	1885	2%	0.94(0.86–1.02)	1.25(1.15–1.36)**
Normal i-PTH and Low Ca and High P	891	1%	2.41(2.15–2.70)**	2.04(1.82–2.29)**
Normal i-PTH and High Ca and Low P	180	0%	2.10(1.71–2.56)**	2.08(1.69–2.57)**
Normal i-PTH and Normal Ca and Low P	1271	1%	1.62(1.49–1.77)**	1.27(1.16–1.38)**
Normal i-PTH and Low Ca and Low P	618	1%	2.94(2.62–3.30)**	1.38(1.22–1.55)**
Normal i-PTH and High Ca and Normal P	4298	5%	0.80(0.75–0.85)**	0.98(0.92–1.05)
Normal i-PTH and Low Ca and Normal P	2691	3%	2.15(2.01–2.31)**	1.38(1.28–1.47)**
High i-PTH and Normal Ca and High P	4336	5%	0.87(0.82–0.93)**	1.08(1.01–1.15)*
High i-PTH and High Ca and High P	5271	6%	0.78(0.74–0.83)**	1.03(0.97–1.09)
High i-PTH and Low Ca and High P	1093	1%	1.89(1.69–2.10)**	1.75(1.57–1.95)**
High i-PTH and High Ca and Low P	69	0%	2.37(1.72–3.28)**	2.24(1.58–3.17)**
High i-PTH and Normal Ca and Low P	326	0%	1.68(1.41–2.01)**	1.29(1.07–1.54)**
High i-PTH and Low Ca and Low P	277	0%	3.60(3.00–4.33)**	1.88(1.55–2.27)**
High i-PTH and High Ca and Normal P	6522	7%	0.73(0.70–0.77)**	0.86(0.81–0.91)**
High i-PTH and Low Ca and Normal P	1590	2%	2.09(1.91–2.28)**	1.46(1.33–1.60)**
Low i-PTH and Normal Ca and High P	2946	3%	1.70(1.59–1.81)**	1.74(1.63–1.85)**
Low i-PTH and High Ca and High P	2152	2%	1.81(1.69–1.94)**	2.06(1.92–2.21)**
Low i-PTH and Low Ca and High P	604	1%	3.04(2.71–3.42)**	2.50(2.22–2.82)**
Low i-PTH and High Ca and Low P	1377	1%	4.29(4.00–4.60)**	2.62(2.43–2.81)**
Low i-PTH and Normal Ca and Low P	4576	5%	2.71(2.58–2.84)**	1.63(1.55–1.72)**
Low i-PTH and Low Ca and Low P	1215	1%	5.28(4.90–5.69)**	1.98(1.83–2.14)**
Low i-PTH and High Ca and Normal P	8394	9%	1.80(1.72–1.88)**	1.78(1.70–1.86)**
Low i-PTH and Low Ca and Normal P	2577	3%	3.28(3.08–3.49)**	1.77(1.66–1.88)**

*P<0.05,

**P<0.01, adjust age, sex, diabetes, albumin, hematocrit, ALK-P and kt/V; i-PTH:<150 Low, 150–300 Normal,≧300 High; Ca:<8.5 Low, 8.5–9.5 Normal, >9.5 High; P:<3.5 Low, 3.5–5.5 Normal, >5.5 High

### All-cause mortality and its associations with intact parathyroid hormone and alkaline phosphatase levels

#### One-year average baseline iPTH or ALP level and 3-year all-cause mortality

**Fig 2:**
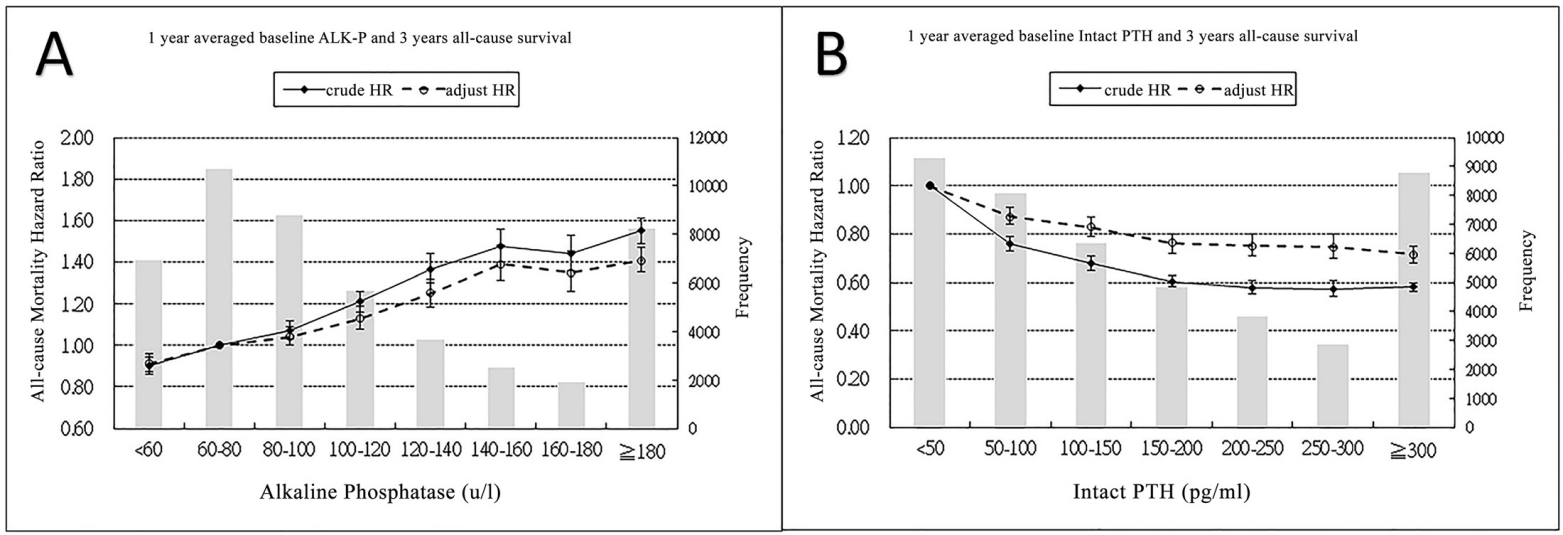
Association between baseline ALP or i-PTH and 3-year all-cause mortality. One-year baseline ALP (A) and one-year baseline i-PTH (B).

Fig 2A illustrates the incremental trends in the three-year crude and adjusted HRs for mortality in ESRD patients in association with higher baseline one-year ALP levels. Fig 2B illustrates that the three-year crude and adjusted HRs for mortality in ESRD patients were higher when the iPTH levels were lower.

#### Time-averaged iPTH or ALP level and all-cause mortality

**Fig 3:**
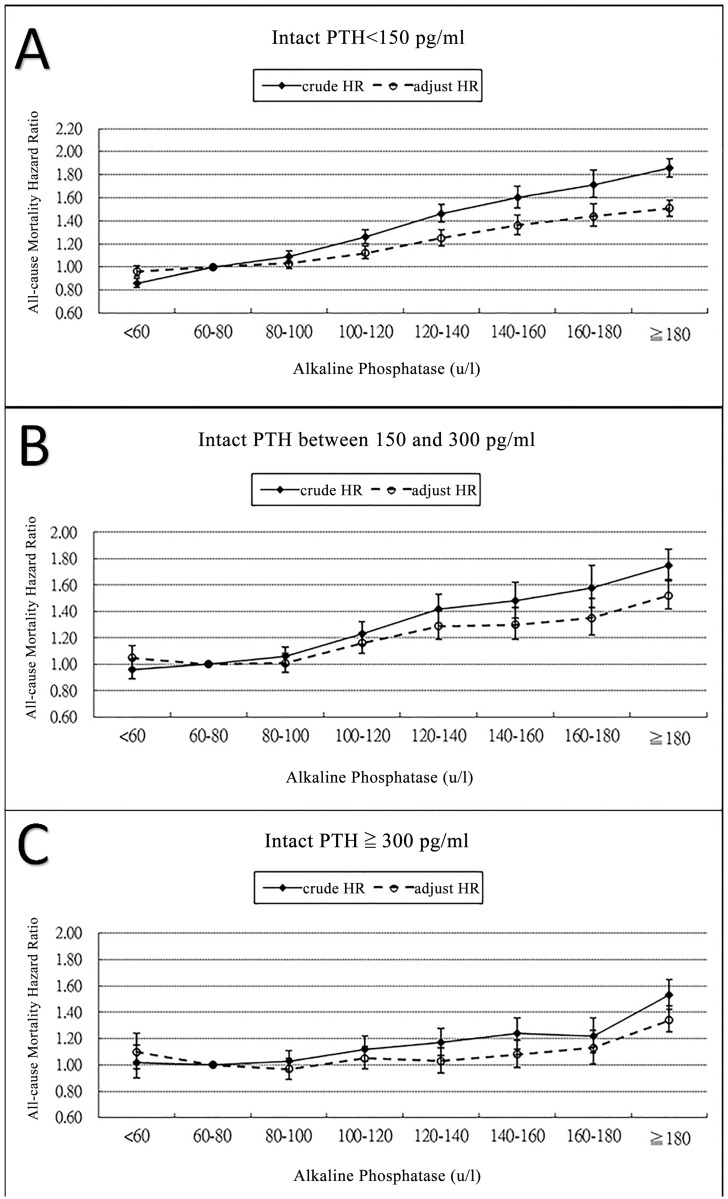
The crude and adjusted all-cause mortality between different groups of i-PTH and time-averaged categories ALP. The i-PTH level was < 150 pg/mL in group A; between 150–300 pg/mL in group B; ≥ 300 pg/mL in group C.

**Fig 4:**
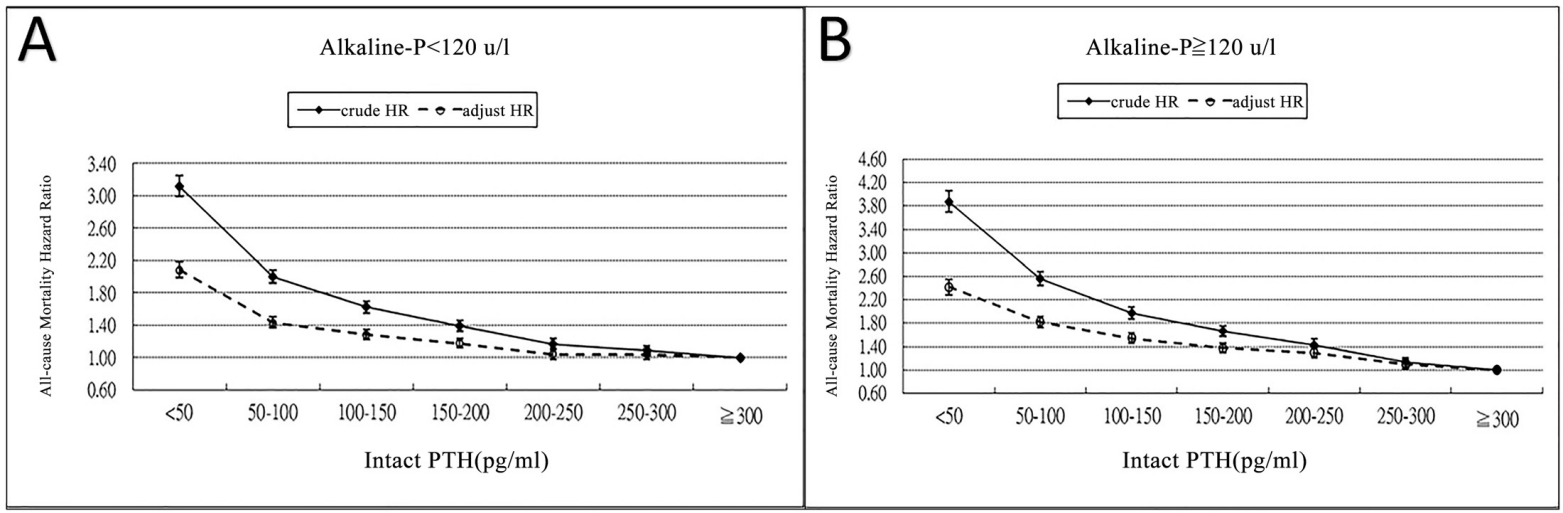
The crude and adjusted all-cause mortality between different groups of ALP and time-averaged categories PTH. The ALP level was < 120 U/L in group A; ≥ 120 U/L in group B.

Fig 3A and 3B illustrates the incremental trends in the crude and adjusted HRs for mortality in ESRD patients in association with the time-averaged ALP levels within the different iPTH categories, especially in patients with low (< 150 pg/mL) (Fig 3A) or normal (150–300 pg/mL) (Fig 3B) iPTH levels. Fig 4A and 4B illustrates that the crude and adjusted HRs for mortality in ESRD patients within the different ALP categories, namely <120 U/L (Fig 4A) and ≥ 120 U/L (Fig 4B), increased when the iPTH levels were lower.

### Calcium and phosphate as predictors of high or low serum intact parathyroid hormone levels

For patients with ALP levels of 50–100 U/L and low iPTH levels, the cumulative HR for the mortality associated with high serum Ca levels (> 9.5 mg/dL) was 1.33 (95% CI 1.27–1.39) (p < 0.01) (Table 4). In contrast, for patients with ALP levels of 50–100 U/L and high iPTH levels, the cumulative HR for the mortality associated with high serum Ca levels (> 9.5 mg/dL) was 0.92 (95% CI 0.85–1.01) (Table 5), a result that was not statistically significant. The same results were obtained for patients with ALP levels of > 100 U/L (data not shown).

**Table 4:** Hazard ratios for death among patients with alkaline phosphatase levels of 50–100 U/L and intact parathyroid hormone levels of < 150 pg/dL.

	Number of patients with data	%	Crude HR (95% confidence interval)	Adjusted HR (95% confidence interval)
**Albumin (g/dL)**				
**< 2.8**	369	2	13.14 (11.70–14.77)**	6.33 (5.56–7.20)**
**2.8–3.6**	6,747	29	2.91 (2.79–3.03)**	2.12 (2.02–2.21)**
**> 3.6**	15,901	69	Reference	Reference
**Hematocrit (%)**				
**< 25**	530	2	4.76 (4.28–5.29)**	2.69 (2.41–3.01)**
**25–30**	7,805	34	1.89 (1.82–1.97)**	1.53 (1.47–1.60)**
**> 30**	14,688	64	Reference	Reference
**Calcium (mg/dL)**				
**< 8.5**	2,063	9	2.02 (1.89–2.16)**	1.31 (1.22–1.40)**
**8.5–9.5**	14,132	62	Reference	Reference
**> 9.5**	6,507	29	1.11 (1.06–1.16)**	1.33 (1.27–1.39)**
**Phosphate (mg/dL)**				
**< 3.5**	3,266	14	1.95 (1.86–2.05)**	1.32 (1.25–1.39)**
**3.5–5.5**	16,522	72	Reference	Reference
**> 5.5**	3,228	14	1.04 (0.97–1.10)	1.17 (1.10–1.25)**

*p < 0.05

**p < 0.01 adjusted for age, sex, diabetes, and the hemodialysis treatment adequacy score, kt/V.

HR: hazard ratio.

**Table 5:** Hazard ratios for death among patients with alkaline phosphatase levels of 50–100 U/L and intact parathyroid hormone levels of > 300 pg/dL.

	Number of patients with data	%	Crude HR(95% confidence interval)	Adjusted HR(95% confidenceinterval)
**Albumin (g/dL)**				
**< 2.8**	30	0	32.85 (21.84–49.40)**	9.55 (6.01–15.20)**
**2.8–3.6**	1,239	13	3.39 (3.01–3.71)**	2.03 (1.83–2.25)**
**> 3.6**	8,348	87	Reference	Reference
**Hematocrit (%)**				
**< 25**	172	2	3.04 (2.41–3.84)**	1.98 (1.55–2.53)**
**25–30**	2,648	28	1.68 (1.55–1.83)**	1.39 (1.27–1.51)**
**> 30**	6,797	71	Reference	Reference
**Calcium (mg/dL)**				
**< 8.5**	1,114	12	2.47 (2.18–2.80)**	1.67(1.46–1.90)**
**8.5–9.5**	4,240	44	Reference	Reference
**> 9.5**	4,182	44	0.76 (0.70–0.83)**	0.92 (0.85–1.01)
**Phosphate (mg/dL)**				
**< 3.5**	169	2	4.03 (3.12–5.22)**	1.96 (1.49–2.58)**
**3.5–5.5**	4,973	52	Reference	Reference
**> 5.5**	4,475	47	1.05 (0.97–1.13)	1.28 (1.18–1.40)**

*p < 0.05

**p < 0.01 adjusted for age, sex, diabetes, and the hemodialysis treatment adequacy score, or kt/V. HR: hazard ratio.

## Discussion

In this large, long-term, nationwide study, approximately 45% of the HD patients had low iPTH levels (< 150 pg/mL). The prevalence of hypoparathyroidism was similar to that reported from a cohort study undertaken in south Asia.[19] However, the frequency of hypoparathyroidism in this study was much higher than the frequencies reported from the Dialysis Outcomes and Practice Patterns Study (DOPPS) that was undertaken in the United States in which 29% of the patients had iPTH levels of < 100 pg/mL,[8] and the United Kingdom Renal Registry (UKRR) study in which 32% of the patients had iPTH levels of < 150 pg/mL. [20] Furthermore, the low iPTH levels did not pose a threat to all-cause mortality in the DOPPS or the UKRR study, which is in contrast to our study’s findings. However, the investigators participating in the UCLA study [21] and the ARNOS study, which was undertaken in France, suggested that low or very low PTH levels (< 50 pg/mL) may correlate with poor survival in HD patients.[22] In addition, the Cox regression analysis showed that higher ALP or lower iPTH levels were associated with higher levels of mortality in HD patients. Of particular interest is that a U-shaped association was determined between PTH levels and the risk of death for dialysis patients in a recent cohort study.[21] As a potential biomarker of CKD-MBD, ALP may be superior compared with PTH because it shows a lower level of intra-individual biological variation.[23] A publication from a Japanese study reported that high ALP levels in patients with low iPTH levels were strongly associated with hip fractures and all-cause or cardiovascular mortality.[24] In this study, we confirmed that high ALP levels were harmful and were associated with mortality in HD patients. Since ALP is expressed in the liver, kidneys, intestines, bones, and leukocytes, high ALP levels may be a marker of inflammation and neutrophil activation.[25] Another publication has suggested that low PTH levels may be associated with inflammation or malnutrition, an association that confounded the relationship between serum PTH and ALP levels.[26] A recent investigation that involved a large sample of American patients who were undergoing HD, demonstrated the importance of controlling PTH levels between 150 pg/mL and 300 pg/mL,[27] but the KDIGO guidelines only recommend regular measurements of ALP and no target range has been specified.

From a recent large cohort study [28], albumin-corrected calcium level over 10.2 mg/dL or phosphate level over 6.2 mg/dL were correlated with higher adjusted mortality. However, in our study, given that high calcium ≥ 9.5 mg/dL and high phosphate ≥ 5.5 mg/dL, mortality was increased only in low iPTH group, but the a phenomenon that was not apparent in patients with normal or high iPTH levels. It is possible that hypercalcemia suppresses PTH secretion, and that its feedback inhibition may provide a beneficial physiological balance in hyperparathyroidism. In addition, the relatively higher values for iPTH levels produced by second-generation iPTH assays compared with those produced by third-generation bio-iPTH assays [29] might mean that the high iPTH levels are overestimations in some patients; hence, hypercalcemia may not be a problem in these patients.

The findings from the Dialysis Outcomes and Practice Patterns Study indicated that serum calcium levels of between 9.6 and 10.0 mg/dL may increase mortality, but statistical significance was not achieved [8]. In this study, we demonstrated that serum calcium levels of more than 9.5 mg/dL will gradually increase mortality (HR 1.05), but the clinical significance was not pronounced until the serum calcium levels reached 10.5 mg/dL (HR 1.77).

Vascular calcification had gained attention because of its associated morbidity and mortality [30], and avoiding Ca loads is crucial to prevent cardiovascular events in HD patients [31]. Most of the studies have focused on high PTH levels, and Ca and P levels and their correlations with mortality in dialysis patients [8]. The findings from our study indicate that high serum Ca levels may be harmful to HD patients with low PTH levels. Two recent meta-analyses of populations with CKD and ESRD determined that higher serum phosphate levels were correlated with higher cardiovascular mortality rates [1,32]; therefore, further investigations into the use of non-calcium-based phosphate binders in HD patients with low iPTH levels are warranted.

No associations were found between high serum Ca levels and increasing mortality in ESRD patients with high iPTH levels in this study. However, in the current study’s population, the patients with high iPTH levels and high P levels but relatively stable Ca levels, were younger and they had the optimal outcomes. Hypercalcemia may also reduce the calcemic effect of PTH, which may benefit secondary hyperparathyroidism.[33] Finally, patients with high PTH levels may have a wider normal range of serum Ca levels because only hyperphosphatemia and the Ca-P product can predict mortality [34].

### Limitations

This study has several limitations that are described next. First, no data were available that described the patients’ body mass indices. However, epidemiologic studies have shown that obese dialysis patients may have better outcomes [35]. Second, when mortality occurred it was recorded as a study drop out, and this occurred because almost all dialysis patients in Taiwan only receive one payment from the government to enable them to use the health care system, hence all patients who died were considered to be study drop outs, except those patients who received renal transplantations. Furthermore, we did not have accurate medical records regarding the use of orally administered calcium-containing phosphate binders, calcimimetics, or vitamin D, but, as yet, no studies have described reductions in mortality associated with the use of these drugs. Finally, the absence of bone specific alkaline phosphatase is also an important limitation in our study, further evaluation and study for bone specific alkaline phosphatase is warranted.

### Conclusions

In this retrospective, large, nationwide, population-based cohort study, we determined that low PTH levels correlated significantly with mortality in HD patients. In addition, low PTH levels and high serum Ca levels were significantly associated with mortality. Additional prospective studies are warranted to investigate the use of non-Ca-containing P binders for the prevention of vascular calcification and complications among uremia patients.
